# The therapeutic value of antidiabetic drugs in lung cancer treatment: from clinical evidence to possible mechanisms

**DOI:** 10.3389/fimmu.2026.1805197

**Published:** 2026-04-29

**Authors:** Qian Wang, Jiayi Xu, Qing Liu, Haixia Zhou, Yue Hu

**Affiliations:** 1 China-Japan Union Hospital of Jilin University, Changchun, China; 2 Department of Radiation Oncology, China-Japan Union Hospital of Jilin University, Changchun, China; 3 Department of Endocrinology and Metabolism, China-Japan Union Hospital of Jilin University, Changchun, China; 4 Department of VIP, China-Japan Union Hospital of Jilin University, Changchun, China; 5 Department of Biobank, China-Japan Union Hospital of Jilin University, Changchun, China

**Keywords:** chemoradiotherapy, diabetes mellitus, hypoglycemic drugs, immunotherapy, lung cancer, targeted therapy

## Abstract

With the steadily rising incidence of lung cancer and diabetes mellitus, both of them are increasingly becoming prominent global health concerns. Treatment options for lung cancer to improve prognosis and reduce the pain and financial burden of patients remain under continuous exploration. In recent years, hypoglycemic agents for diabetes mellitus have gained growing recognition for their potential anti-tumor effects through multi-pathway regulation, particularly in combination therapies, where they may enhance the efficacy of traditional chemotherapy or targeted treatments for lung cancers. Therefore, in-depth investigation into the effects of various hypoglycemic agents on lung cancer, along with their underlying mechanisms, is of great significance for developing more precise and effective treatment strategies, optimizing therapeutic outcomes, and improving patient survival rates. In the present review, we will summarize the recent progress of whether and how hypoglycemic agents could be potential complementary options for lung cancer treatment and explore the possible underlying mechanism. While examining the pharmacological profile of metformin relevant with lung cancer treatment, this review also explores the unique idiosyncrasies of other commonly used agents in diabetes mellitus management.

## 1 Introduction

Lung cancer (LC), characterized by its aggressive nature, ranks as the second most prevalent cancer globally, constituting 11.4% of all cancer cases. It is also the foremost cause of cancer-related fatalities, accounting for 18% of cancer mortality rates (1, 2), representing a major threat to human health. Lung cancer is histologically classified according to the WHO system, primarily into non−small cell lung cancer (NSCLC) and small cell lung cancer (SCLC). NSCLC accounts for 80%–85% of all lung cancer cases, while SCLC accounts for 15%–20% (3). The initiation and progression of lung cancer are driven by the dysregulation of multiple signaling pathways. Current clinical and translational research focuses mainly on the epidermal growth factor receptor (EGFR), anaplastic lymphoma kinase (ALK), and programmed cell death protein 1 (PD−1)/programmed cell death ligand 1 (PD−L1) pathways, as well as genetic alterations including ROS1 fusion, BRAF mutation, and NTRK fusion. Lung cancer treatment has entered an individualized era of precision and immunotherapy, with core modalities including surgery, radiotherapy, chemotherapy, targeted therapy, and immunotherapy (4). Surgery is first-line radical therapy for stage I–II NSCLC. Radiotherapy is indicated for radical treatment in early inoperable cases, concurrent chemoradiotherapy for locally advanced disease, palliative care for advanced disease, and brain metastasis management. Combination chemotherapy remains the backbone of advanced NSCLC treatment. Targeted therapies against driver mutations (e.g. EGFR inhibitors osimertinib/gefitinib, ALK inhibitors crizotinib/alectinib) significantly prolong progression-free survival (PFS), while PD-1/PD-L1 blockade-based immunotherapy further improves clinical outcomes (5). Overall, lung cancer prognosis remains poor, mainly determined by pathological stage, histological subtype, driver gene status, and treatment response, with NSCLC 5-year survival rates of 60%–80% (stage I), 30%–50% (stage II), 10%–20% (stage III), and only 5%–10% (stage IV), highlighting unmet needs for novel adjuvant or combinatorial strategies (6).

The relationship between diabetes mellitus (DM) and LC has long been a topic of debate in epidemiological and clinical research. The weak association between diabetes and lung cancer observed in previous studies is essentially the effect of confounding factors such as smoking, obesity and chronic obstructive pulmonary disease, rather than a direct effect of diabetes itself (6). Studies indicate that the incidence of malignant tumors in patients with T2DM reaches 28.35%, with digestive tract tumors, hematological malignancies, and lung cancer among the most common (7). Diabetes provides a nutrient-rich microenvironment for lung cancer cells through metabolic disorders (hyperglycemia/hyperinsulinemia/dyslipidemia), and induces oxidative stress and DNA damage via chronic inflammation (TNF-α/IL-6/leptin). Together, these factors activate oncogenic signaling pathways such as NF-κB, JAK/STAT3 and PI3K/Akt/mTOR, driving metabolic reprogramming, malignant proliferation, invasion and metastasis of lung cancer cells (8, 9).

Increasing evidences suggest that conventional glucose-lowering agents such as insulin, insulin sensitizers, and secretagogues may influence the risk of cancer, including lung cancer (10). However, conclusive evidence linking specific hypoglycemic agents to direct carcinogenic effects is still insufficient. The relationship between various hypoglycemic agents and the development of malignant tumors has gained increasing attention. Some clinical studies suggest that hypoglycemic therapies associated with hyperinsulinemia, such as sulfonylureas and exogenous insulin, may elevate cancer risk, while drugs that improve insulin resistance, like metformin, could potentially reduce this risk (11, 12). The mechanisms through which hypoglycemic agents, including insulin, insulin sensitizers, and secretagogues, impact tumor development remain to be fully understood. According to their mechanisms of action and indications, hypoglycemic agents can be classified into several major categories: metformin, sulfonylurea drugs, insulin, thiazolidinediones, incretins, and SGLT-2 Inhibitors. The present review seeks to elucidate the intricate relationship between antidiabetic therapies and the clinical outcomes associated with LC.

## 2 Metformin in lung cancer treatment

Metformin, an oral hypoglycemic agent, has shown excellent effectiveness in lowering fasting blood glucose levels of DM patients. This medication has been utilized in clinical settings for over six decades, following its introduction in 1957 (13). Metformin serves as the primary therapeutic intervention for type 2 diabetes mellitus (T2DM), primarily functioning to reduce glucose levels through the activation of the AMPK pathway (14). Recent experimental studies have demonstrated that metformin not only suppresses the proliferation of tumor cells but also enhances the sensitivity of tumors to chemotherapy and small-molecule targeted anticancer agents (15). Metformin has been demonstrated in preclinical studies to induce cell cycle arrest in various cancer cell lines, including those from the breast, kidney, brain, ovary, lung, and endometrium, thereby inhibiting tumor cell proliferation (16–18).

### 2.1 Mechanisms of metformin on lung cancer treatment

**Figure 1:**
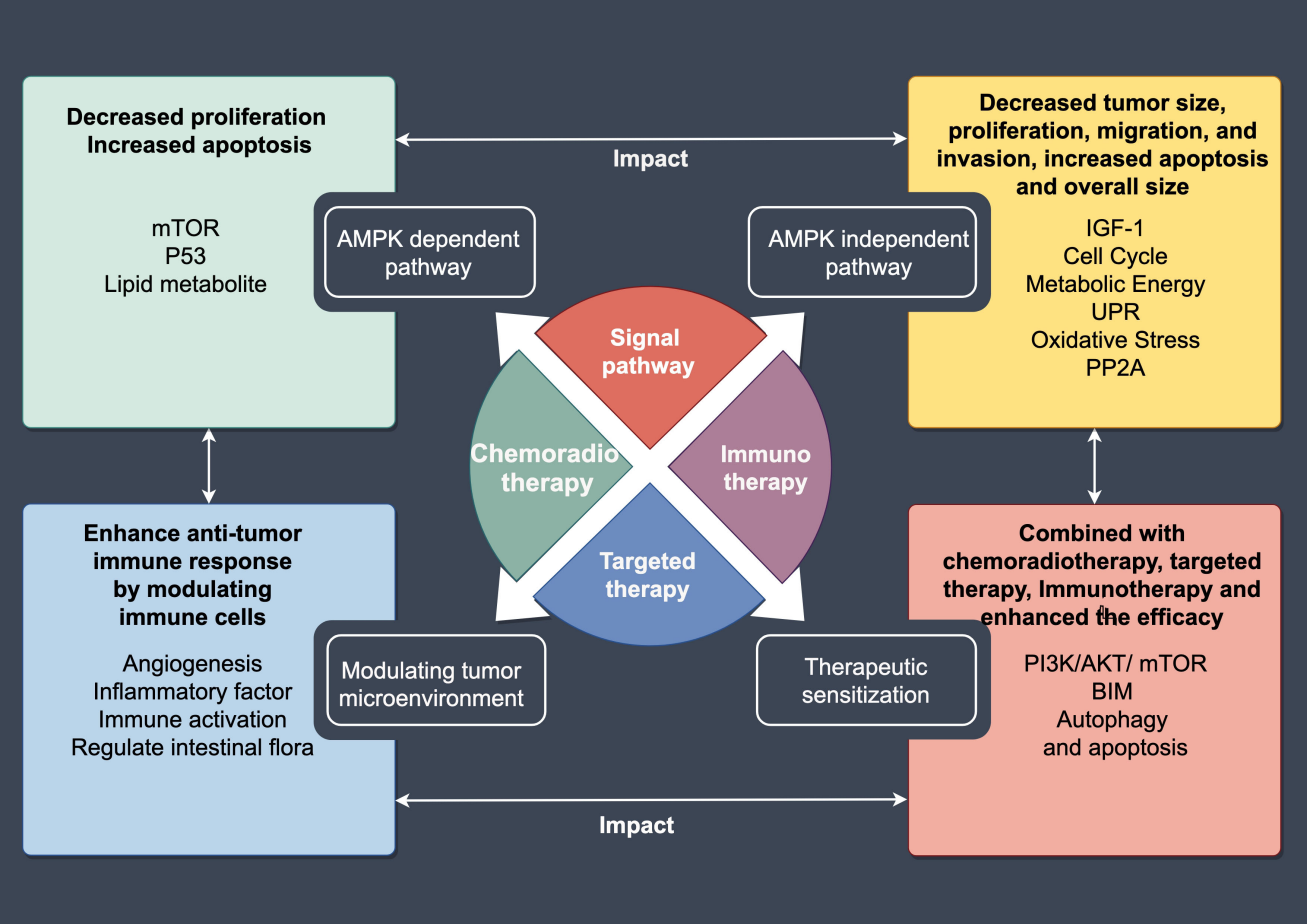
A summary diagram of the mechanism of action of metformin.

Metformin benefits in tumor treatment mainly through multiple pathways: (a) Activating the adenosine monophosphate-activated protein kinase (AMPK) pathway, inhibiting mammalian target of rapamycin (mTOR), and reducing tumor cell proliferation; (b) Inducing apoptosis of tumor cells and regulating the cell cycle; (c) Enhancing the sensitivity to radiotherapy and chemotherapy; (d) Inhibiting tumor angiogenesis, improving the tumor microenvironment and work together with immunotherapy to exert an anti-tumor effect (**Figure 1**). Metformin induces programmed cell death by activating the AMPK/liver kinase B1 (LKB1)/target of rapamycin complex 1 (TORC1) signaling cascade, consequently suppressing mTOR activity. The inhibition of mTOR activity can lead to cell growth arrest and reduced protein synthesis, thereby suppressing the proliferation of tumor cells (19–21). The activation of AMPK affects the cell cycle protein-dependent kinase (CDK) inhibitory factors p53 and p21, causing the cell cycle to remain in the G1 or G2 phase, preventing cells from entering the mitotic stage, thereby inhibiting the proliferation of tumor cells (22). The activation of AMPK can promote the release of cytochrome C by mitochondria, activate caspase family proteins, and induce apoptosis (23). Metformin also increases tumor radiosensitivity by inhibiting mitochondrial oxygen consumption, thereby enhancing tumor oxygenation. Metformin further increases radiosensitivity by downregulating the EGFR/PI3K/Akt pathway, cell cycle arrest, and reduced colony formation (24, 25). It also reverses epithelial-to-mesenchymal transition (EMT) in human lung cancer cell lines with EGFR mutations, significantly inhibiting the activation phosphorylation of extracellular signal-regulated kinase (ERK)1/2 and p70S6K, while upregulating E-cadherin expression (26).

In metabolic reprogramming, metformin can inhibit the glycolysis process of tumor cells, reduce the production of lactic acid, and lower the energy supply of tumor cells (27). Metformin reduces the degree of hypoxia in the tumor microenvironment by inhibiting the release of pro-inflammatory factors such as IL-6 and TNF-α (28). Moreover, metformin can activate innate immune cells, such as macrophages and natural killer cells, and enhance their phagocytic and killing effects on tumor cells. Metformin stimulates AMPK, which results in the destabilization of hypoxia-inducible factor 1α (HIF-1α), and consequently lowers the expression of vascular endothelial growth factor (VEGF). The modulation of angiogenesis contributes to the enhancement of the tumor microenvironment (29, 30).

### 2.2 Clinical investigations of metformin on lung cancer treatment

Numerous clinical investigations have demonstrated that administration of metformin correlates with a diminished likelihood of developing LC (31). A meta-analysis examining LC revealed that patients with T2DM who were treated with metformin exhibited a lower risk of developing LC in contrast to those receiving other standard medications. Yao et al. (32) conducted a meta-analysis which included 10 cohort studies and 3 case-control studies and found that individuals using metformin exhibited an 11% reduction in the risk of developing LC compared to those who did not use the medication (RR = 0.89, 95% CI: 0.83-0.96, P = 0.002). Katherina et al. (33) conducted a study with a substantial sample size of 732,199 participants from a national health examination in South Korea from the year of 2002 to 2003. Among patients with DM, those with cumulative metformin use of ≥1.5 years exhibited a significantly lower incidence of lung cancer (aRR = 0.44) and a reduced lung cancer-related mortality rate (aRR = 0.76) compared with DM patients who did not receive metformin. Further analysis revealed a dose-dependent negative correlation between metformin use and both lung cancer incidence and mortality in the DM population. Key characteristics of the above clinical studies are summarized in **Table 1**.

**Table 1:** Main characteristics of clinical studies.

Therapy class	Year	Study region	Sample size	Histology	Treatment strategy	Dose of hypoglycemic drug	Synergistic effect
Chemo therapy	2015	Egypt	30	NSCLC	Gemcitabine/cisplatin	500mg daily	The OS in the metformin group was 12 months versus 6.5 months in patients not treated with metformin, *p*=0.119. The PFS was 5.5 months versus 5 months, *p*=0.062.
	2015	US	750	NSCLC	Not describe	Not describe	The OS in the metformin group was 5 months versus 3 months in patients not treated with metformin, *p*< 0.001.
	2015	China	259	SCLC	Not describe	Not describe	The OS in the metformin group was 19 months versus 11.5 months in patients not treated with metformin, p < 0.001. The PFS in the metformin group was 10.5 months versus 7 months in patients not treated with metformin, *p* < 0.001.
	2015	China	79	SCLC	Etoposide+ platinum Irinotecan+ platinum	Not describe	The OS in the metformin group was 18 months versus 11.5 months in patients not treated with metformin, *p *< 0.001. The PFS in the metformin group was 10.8 months versus 6.5 months in patients not treated with metformin, *p* < 0.001.
	2017	US	14	NSCLC	Carboplatin+pemetrexed	1000 mg/day for week 1, 1500 mg/day for week 2, then 2000 mg/day	NS
	2018	China	75	NSCLC	Platinum-based	Not describe	NS
Chemo radio therapy	2016	Netherlands	682	NSCLC	Concurrent chemoradiotherapy	Not describe	The DMFS was74% versus 53% at 2years and PFS was 58% versus 37% at 2 years
	2015	US	166	NSCLC	Concurrent chemoradiotherapy	Not describe	NS
	2021	US	170	NSCLC	Carboplatin and paclitaxel	Increasing from 1,000 mg/day in week 1 to 2,000 mg/day over 2 weeks	NS
Targeted therapy	2015	China	90	NSCLC	EGFR-TKIs	Not describe	The OS in the metformin group was 19 months versus 8 months in patients not treated with metformin, *p* = .005. The PFS in the metformin group was 32 months versus 23 months in patients not treated with metformin, *p* = .002.
	2019	China	1633	NSCLC	EGFR-TKIs (gefitinib and erlotinib)	Not describe	Metformin use was associated with a significantly longer median PFS (9.2 months, *p* <.001) and OS (33.4 months, *p* <.001)
	2016	Mexico	1106	NSCLC	EGFR-TKIs	Not describe	The OS in the metformin group was 25.6 vs 13.2 months in patients not treated with metformin, *p* = 0.017 The OS in the metformin group was 40.5 vs 13.2 in patients not treated with metformin, *p* < 0.001
Immuo therapy	2018	Japan	–	Solid tumor	Nivolumab	Started at 750 mg/day	(NCT03048500)
	2019	US	50	NSCLC	Anti-PD-1 (pembrolizumab, nivolumab), Anti-PD-L1 (atezolizumab)	500 mg twice daily	The OS was 11.5 in the metformin group vs 7.6 months in patients not treated with metformin, *p* = 0.5 The PFS was 4.0 in the metformin group vs 3.0 months in patients not treated with metformin, *p* = 0.6

#### 2.2.1 Metformin and chemotherapy

Numerous studies indicate that metformin has been shown to reduce or reverse multidrug resistance in tumors through various mechanisms (34–36). A prospective, randomized, open-label, controlled pilot study was carried out involving patients diagnosed with stage IV NSCLC (37). Subjects were allocated randomly in a 1:1 ratio to receive either a combination of gemcitabine and cisplatin alongside metformin therapy or gemcitabine and cisplatin chemotherapy respectively, with metformin dosed at 500 mg per day. The observed response rates to the treatment were 46.7% in the metformin cohort, whereas the cohort without metformin exhibited a rate of 13.3%. The OS was observed to be 12 months compared to 6.5 months, while the PFS was noted as 5.5 months in contrast to 5 months, respectively. Ahmed (38) carried out a retrospective cohort study at a single institution, focusing on patients with NSCLC who received chemoradiation treatment. Among the cohort of 40 individuals diagnosed with type II diabetes, half, specifically 20 participants, were administered metformin, while the remaining 20 did not receive this treatment. No notable differences in survival or failure patterns were detected among these patients. Kong et al. (39) investigated the potential prognostic advantages of metformin administration in patients with SCLC. 259 patients with diabetes and SCLC were included in the study. The OS and disease-free survival (DFS) were significantly improved in the metformin cohort compared to the non-metformin cohort (OS 19.0 vs 11.5 months, p < 0.001; DFS 10.5 vs 7.0 months, p < 0.001). Comparable results were documented by Xu et al. (40) suggesting that the prognosis for SCLC patients with diabetes who also received metformin was notably improved. A study involving 79 patients with SCLC demonstrated a significant improvement in OS and DFS among those treated with metformin, compared to those who did not receive the medication. (OS 18.0 versus 11.5 months, p < 0.001; disease-free survival 10.8 versus 6.5 months, p < 0.001).

Nonetheless, numerous investigations also have indicated that adjuvant metformin therapy does not exert a significant effect on survival outcomes. For example, Parikh et al. carried out a prospective phase II clinical trial that included 14 patients diagnosed with stage III or IV LC, who were administered carboplatin, pemetrexed, and metformin. The research findings indicate that incorporating metformin into chemotherapy regimens for advanced NSCLC is deemed safe; however, it does not yield a notable enhancement in clinical efficacy when juxtaposed with earlier phase III trials (41). A further investigation involving 75 patients with NSCLC and T2DM evaluated the survival outcomes of those undergoing platinum-based chemotherapy while receiving metformin. The average OS was recorded at 36.74 months for the metformin cohort and 40.21 months for the non-metformin cohort, revealing no statistically significant difference in OS (P = 0.661) (42).

Concurrent chemoradiotherapy is essential in the management of locally advanced NSCLC and limited-stage SCLC. The interplay between metformin and concurrent chemoradiotherapy continues to provoke discussion within the scientific community. In a retrospective analysis involving 682 patients diagnosed with locally advanced NSCLC (43), 59 individuals were administered metformin alongside chemoradiotherapy, whereas 623 did not receive this treatment. The results indicated while metformin did not have a notable impact on local relapse-free survival (LRFS) and OS; nonetheless, it demonstrated an enhancement in distant metastasis-free survival (DMFS) and PFS, prolonging the duration without tumor progression by an average of 26 months. Another study conducted by the Rutgers Cancer Institute of New Jersey, which examined diabetic patients with or without metformin treatment, revealed comparable outcomes in OS (14.3 vs 19.2 months, P = 0.18), PFS (19.7 vs 10.1 months, P = 0.38), locoregional recurrence-free survival (RFS) (11.9 vs 15.5 months, P = 0.69), and DMFS (10.0 vs 17.4 months, P = 0.12) across the two cohorts (37). The NRG-LU001 study, an open-label phase 2 trial, involved 170 non-diabetic patients diagnosed with unresectable stage III NSCLC. These patients received carboplatin- and paclitaxel-based chemoradiotherapy, either as monotherapy or combined with metformin, with the metformin dose escalating from 1,000 mg/day in the first week to 2,000 mg/day by the second week. While the metformin group exhibited favorable tolerance, there were no notable differences in survival rates, local recurrence, or distant metastasis when compared to the control group (44).

#### 2.2.2 Metformin and targeted therapy

In individuals diagnosed with EGFR-mutated NSCLC, the synergistic application of metformin alongside EGFR-TKIs has shown improved therapeutic outcomes. A retrospective analysis of 90 patients from six hospitals in China who were treated with EGFR-TKIs and metformin found that diabetic NSCLC patients (n = 44) experienced significantly improved PFS and OS compared to those receiving EGFR-TKIs with other hypoglycemic agents (n = 46) (19.0 months vs 8.0 months, P = 0.005; 32.0 months vs 23.0 months, P = 0.002) (45). A cohort study conducted in Taiwan indicated that metformin might enhance survival rates in diabetic LC patients receiving EGFR-TKI therapy, which includes gefitinib and erlotinib. These findings revealed that the use of metformin was correlated with a notably extended median PFS of 9.2 months (95% CI: 8.6-11.7) compared to 6.4 months (95% CI: 5.9-7.2), with a statistically significant difference (P <.001). Additionally, OS was also improved, with a median of 33.4 months (95% CI: 29.4-40.2) versus 25.4 months (95% CI: 23.7-27.2), again showing significant results (P <.001) (46). Cardona et al. conducted an analysis on how diabetes and its treatment influenced the survival rates of patients diagnosed with LC. In summary, individuals with diabetes who were administered metformin demonstrated a significantly enhanced OS when contrasted with those receiving alternative antidiabetic therapies (25.6 months versus 13.2 months, p = 0.017). Individuals exhibiting controlled glycemic levels demonstrated superior OS compared to those with unmanaged diabetes and non-diabetic counterparts, with durations of 40.5 months, 13.2 months, and 18.5 months, respectively (p < 0.001) (47). The results indicate that metformin may improve the therapeutic efficacy of EGFR-TKI in individuals with T2DM.

Research has explored the synergistic impacts of gefitinib and metformin on NSCLC (48). This combination therapy demonstrated notable effects on inhibiting cell proliferation and promoting apoptosis, with the percentage of apoptotic cells varying between 65-78%. In contrast, gefitinib alone resulted in apoptotic cell percentages of 4-19%, while metformin alone yielded 5-21%. Gefitinib resistance presents a prevalent challenge, manifesting as either primary resistance, characterized by a lack of response or disease progression within three months, or as acquired resistance following an initial therapeutic response (48, 49). Intrinsic resistance is linked to some elements, including the insulin-like growth factor-1 receptor (IGF-1R) receptor, the BIM mutation, and the insulin-like growth factor-1 receptor. According to Pan et al., metformin may make primary resistant NSCLC cells more sensitive to gefitinib, particularly IGF-1R dependent cells, which show higher activation levels of IGF-1R in EGFR-TKI resistant cells (49). The blockade of IGF-1R through AG-1024 or siRNA resulted in an amplification of gefitinib’s efficacy, leading to increased apoptosis and diminished proliferation. Similarly, the combination of metformin and gefitinib inhibited tumor growth by suppressing AKT signaling and upregulating BIM, thereby enhancing the sensitivity of resistant cells (49).

Rearrangements in the ALK gene are found in approximately 3%-7% of NSCLC patients (50), making them suitable candidates for therapy with ALK tyrosine kinase inhibitors. One effective medication for ALK-rearranged NSCLC is crizotinib, a first-generation ALK tyrosine kinase inhibitor (50). Nonetheless, the majority of patients exhibited resistance to crizotinib within a year of starting treatment (51, 52). Li et al. demonstrated that metformin increased crizotinib sensitivity in resistant NSCLC cells by inhibiting the IGF-1R pathway, thereby restoring their responsiveness to the drug (53). Metformin and crizotinib worked together to increase apoptosis rates while decreasing cell invasion and proliferation. Metformin attenuated crizotinib resistance by decreasing IGF-1R signaling and phosphorylation of mTOR, p70S6K, and S6, although it has no effect on AKT activation.

The research additionally examined the potential of metformin to mitigate hepatocyte growth factor (HGF)-induced resistance to alectinib, which is classified as another ALK-TKI (54). Metformin did not directly inhibit mesenchymal–epithelial transition (MET) activation but significantly suppressed downstream HGF/MET signaling, leading to reduced phosphorylation of key proteins such as AKT, mTOR, ERK, and p70S6K. This combinational approach markedly enhanced antitumor activity against alectinib-resistant NSCLC xenografts in mouse models (54).

#### 2.2.3 Metformin and immunotherapy

Recent studies have confirmed that metformin can significantly potentiate the anti-tumor immune response in the body, and this effect is primarily achieved through the precise regulation of immune cell function and the remodeling of metabolic and immune characteristics of the tumor microenvironment (TME). Metformin breaks the immunosuppressive barrier of the TME through a dual approach of metabolic reprogramming and immune activation.

In terms of TME metabolic regulation, metformin activates AMPK by inhibiting mitochondrial complex I. On the one hand, it blocks the Warburg effect in tumor cells, reverses their abnormal aerobic glycolysis pattern, reduces the accumulation of metabolic waste such as lactic acid in the TME, and ameliorates the immune cell dysfunction induced by the acidic microenvironment (55). On the other hand, it inhibits tumor cell anabolism through the AMPK-mTOR pathway, decreases the circulating levels of insulin/insulin-like growth factor 1 (IGF-1), and blocks the phosphatidylinositol 3-kinase (PI3K)/protein kinase B (Akt)/mTOR pro-tumor signaling pathway (56). Meanwhile, it restores the expression of Caveolin-1 in cancer-associated fibroblasts, inhibits nutrient supply from stromal cells to tumor cells, and suppresses tumor progression from both metabolic and nutritional supply perspectives. In terms of immune microenvironment activation, metformin can remodel the TME from an immunosuppressive “cold tumor” to an immune-activated “hot tumor”. It achieves the balanced regulation of immunosuppressive cells and effector cells by promoting the polarization of tumor-associated macrophages (TAMs) from the pro-tumor M2 phenotype to the anti-tumor M1 phenotype, enhancing the cytotoxic activity of CD8^+^ cytotoxic T cells and natural killer (NK) cells, and inhibiting the proliferation and immunosuppressive function of regulatory T cells (Tregs) and myeloid-derived suppressor cells (MDSCs) (57). A study conducted at the University of Helsinki, metformin helps dendritic cells mature by blocking mitochondrial complex I. As a result, CD4^+^ T cell proliferation and activation are both improved, which strengthens anti-tumor immunity (58). The tumor microenvironment, known for its immunosuppressive nature, poses significant challenges to CD8^+^ T cells in mounting effective anti-tumor responses. Some studies indicated that metformin might elevate the population of CD8^+^ tumor-infiltrating lymphocytes (TILs), inhibit PD-1 gene transcription, and bolster the anti-tumor activity of CD8^+^ T cells (59). Furthermore, metformin has demonstrated the ability to reduce tumor hypoxia, thereby enhancing the functionality of CD8^+^ T cells (60).

A retrospective analysis of 50 patients with stage IV NSCLC undergoing ICIs (immune checkpoint inhibitors) as part of combination therapy or in second- or third-line treatment indicated that the overall response rate and disease control rate were elevated in the metformin-ICI cohort (41.1% vs 30.7%, p = 0.4 and 70.5% vs 61.6%, p = 0.5, respectively). The median OS and PFS were observed to be elevated in the metformin-ICI cohort, with values of 11.5 months compared to 7.6 months (p = 0.5) and 4.0 months versus 3.0 months (p = 0.6), respectively (61). A current phase II clinical trial (NCT03048500) is investigating the benefits, safety, and tolerability of the combination of nivolumab and metformin in patients with unresectable stage III-IV NSCLC who are immunotherapy-naive as well as those who have received prior treatment (62). Nevertheless, attaining optimal therapeutic plasma levels of metformin presents significant challenges, as elevated doses could pose toxicity risks to humans. Future clinical trials ought to concentrate on refining regimens that utilize reduced metformin concentrations to assess its anti-cancer properties with greater efficacy.

## 3 Sulfonylurea hypoglycemic drugs

Sulfonylureas, like metformin, are well-established hypoglycemic agents that have been used clinically for many years. Their mechanism involves stimulating insulin secretion from pancreatic beta cells. Sulfonylureas specifically interact with ATP-sensitive potassium channels located on the membrane of beta cells. This interaction facilitates the influx of calcium, leading to elevated intracellular calcium concentrations that ultimately stimulate the release of insulin. As a result, the administration of sulfonylureas could induce hyperinsulinemia, which may elevate the likelihood of cancer development. Nonetheless, the existing studies have yet to clearly clarify the impact of these medications on the risk of developing cancer. Ruiter et al. (63) observed that individuals undergoing metformin therapy demonstrated a reduced overall risk of developing a range of cancers, such as esophageal, gastric, colorectal, liver, pancreatic, respiratory tract, breast, and prostate cancers, in comparison to those receiving sulfonylureas (HR = 0.90, 95% CI: 0.88-0.91). A British cohort study involving 52,600 diabetic patients utilizing sulfonylureas revealed an increased risk of malignancies, correlated with both the duration and dosage of sulfonylurea administration (64). High sulfonylurea receptor1 (SUR1) expression is upregulated in NSCLC tissues and cell lines. Targeting SUR1 with glibenclamide suppressed cell proliferation, cell-cycle progression, epithelial–mesenchymal transition (EMT), and cell migration by inhibiting the SUR1/p70S6K pathway (65). In the indirect effect pathway, high SUR1 expression in tumor tissues was positively correlated with α−SMA positive staining of cancer-associated fibroblasts (CAFs) and with poor prognosis in NSCLC patients. Glibenclamide, which targets SUR1, suppressed CAF activation and inhibited tumor growth in patient-derived xenograft models (66).

## 4 Insulin and its analogues

When oral hypoglycemic agents are not effective enough in controlling high blood glucose levels, insulin therapy is usually used. In order to achieve adequate glycemic control, insulin is necessary for about 50% of people with T2DM (67). Endogenous vs. exogenous insulin explains conflicting lung cancer risk findings. Endogenous hyperinsulinemia, mostly resulting from insulin resistance, promotes tumorigenesis and progression through the cell proliferation and angiogenesis signaling pathways mediated by the insulin receptor (IR)/IGF-1R. A clinical study involving 37 patients with EGFR-mutant advanced NSCLC demonstrated that hyperinsulinemia (fasting insulin level ≥32.24 μIU/mL) was an independent risk factor for poor prognosis in these patients (HR = 2.803, 95% CI: 1.063–7.393) (68). In contrast, exogenous insulin effectively improves glycemic control without inducing sustained hyperinsulinemia. A meta-analysis of 42 observational studies showed that exogenous insulin exerted a neutral effect on the risk of most malignancies, including lung cancer (69).

The ability of IGF-1 and IGF-1R to enhance angiogenesis is due to activating VEGF gene transcription (70, 71). One promising target for cancer treatment is IGF-1R, since regulating its expression in tumor cells may inhibit cell proliferation and induce cell death. Additionally, it is possible that enhanced cell proliferation and the development of LC are outcomes of the indirect effects IGF-1R activation on EGFR pathways. Evidence from immunohistochemistry shows that patients with NSCLC who have an overabundance of IGF-1R have a reduced response to gefitinib treatment. The fact that IGF-1R is upregulated in response to EGFR -TKI suggests that it may play a role in the main mechanism of TKI resistance (72). Activation of IGF-1R in NSCLC cell lines reduces the anticancer effects of erlotinib by increasing the number of EGFR/IGF-1R heterodimers localized to the cell membrane, which in turn boosts the production of survivin, a protein that prevents cell death. Resistance to EGFR inhibition, which is mediated by IGF-1R, develops in NSCLC due in large part to the interaction between EGFR and IGF-1R signaling (73). At first, EGFR TKI seems to be helpful for many NSCLC patients who have EGFR activating mutations, like exon 19 deletions or L858R mutations. Nevertheless, a considerable portion of patients eventually gain resistance to these medications, frequently due to additional mutations, and the IGF-1R route bypassing the EGFR signaling pathways (74). A case-cohort study by Mondul et al. investigated the association between serum insulin levels and the risk of LC. The study included 196 cases and 395 subcohort members, and it suggested that targeting IGF-1R or related bypass mechanisms could be an effective way to tackle the therapeutic challenges faced in LC treatment. The study also found that the homeostatic model assessment for insulin resistance (HOMA-IR) was positively associated with the risk of lung cancer (HR = 1.83, 95% CI: 0.99–3.38), further supporting the carcinogenic effect of endogenous insulin disorders. Compared to patients in the lowest insulin quartile, those in the highest insulin quartile had a significantly higher risk of developing LC (HR = 2.10, 95% CI = 1.12-3.94). A higher risk of developing LC has been linked to elevated insulin levels and insulin resistance (75).

## 5 Other hypoglycemic agents

### 5.1 Thiazolidinediones

TZDs are hypoglycemic agents that operate through the activation of peroxisome proliferator-activated receptor gamma (PPARγ), which increases tissue sensitivity to insulin and consequently produces a glucose-lowering effect. Rangaswany et al. reported in a cohort of veterans that the utilization of TZD was associated with a 33% reduction in LC risk among diabetic individuals when compared to those who did not use the medication, following adjustments for confounding variables (HR 0.67, 95% CI: 0.51-0.87) (76). *In vitro* investigations have demonstrated that PPARγ activators can trigger cell cycle arrest and apoptosis in LC cell lines (77, 78). PPARγ exhibits elevated expression levels in vascular endothelial cells associated with tumors; however, its expression diminishes swiftly following TZD exposure, indicating that the inhibition of angiogenesis could play a crucial role in the anti-cancer effects of TZDs (79). Research indicates that the anti-tumor mechanisms of TZDs might parallel those of metformin, involving improvement in insulin sensitivity, inhibition of mitochondrial oxidative phosphorylation, and activation of the AMPK pathway (80).

### 5.2 Incretins

A novel class of medications known as incretins includes glucagon-like peptide-1 (GLP-1) receptor agonists and dipeptidyl peptidase-4 (DPP-4) inhibitors, both of which reduce blood glucose levels. Incretins could inhibit glucagon release, delay stomach emptying and reduce appetite. Their primary mechanisms of action include promoting β-cell proliferation while simultaneously preventing apoptosis. They also stimulate glucose-dependent insulin production from pancreatic beta cells.

Wesley et al. reported a significant reduction in both mRNA and protein expression levels of the DPP-4 gene in NSCLC cells compared to normal lung tissue, with complete absence observed in some cases (81). Restoration of DPP-4 expression significantly altered tumor cell morphology, suppressed tumor formation, metastasis, and proliferation in nude mice, and promoted P21 expression, thereby inducing apoptosis and causing cell cycle arrest at the G1 phase (81). Inhibition of DPP-4 enhances anti-tumor immunity through two key mechanisms. First, it recruits eosinophils—critical for tumor cell apoptosis and amplifying anti-tumor immune responses in the TME—by preserving chemokines like C-C motif chemokine ligand 11 (CCL11) and synergizes with immune checkpoint therapy, representing a novel eosinophil-mediated anti-tumor pathway (82). Second, DPP-4 inhibition can also enhance anti-tumor immune responses by improving the functionality of type 1 conventional dendritic cells (cDC1s). Sitagliptin, for example, boosts cDC1s’ antigen-presenting capacity to facilitate T cell activation and tumor suppression. Mechanistically, it stabilizes key signaling molecules for cDC1s and restores TME glucose availability to meet the metabolic requirements of these immune cells (83). A recent meta-analysis of randomized controlled trials revealed no significant correlation between DPP-4 inhibitors and the risk of LC (combined relative risk 1.00, 95% CI: 0.76-1.33) (84). The SAVOR-TIMI 53 trial, which included a cohort of 16,492 patients, indicated that saxagliptin was associated with a reduced incidence of LC cases in comparison to the placebo group (54/326 vs 59/362; HR 0.91, 95% CI: 0.63-1.32) over a median follow-up period of 2.1 years (85).

GLP-1 receptor agonists, such as the short-acting exenatide and the long-acting liraglutide, enhance insulin secretion and inhibit glucagon release. Effective in the treatment of T2DM and obesity, these agents also enhance satiety, reduce food intake, and delay gastric emptying (86). Liraglutide exhibits the capacity to impede the proliferation, cell cycle progression, and migration of LC cells. It additionally diminishes the senescence of lung cells and mitigates stress within the endoplasmic reticulum (87). Genetic evidence further clarifies the role of the GLP-1 signaling pathway in tumor immunology: Zhu et al. analyzed GLP-1 signaling-related genes across 33 cancer types and identified a significant association between the GLP-1 signaling pathway and immune cell infiltration levels, including CD4^+^ T cells, natural killer (NK) cells, neutrophils, dendritic cells (DCs), and macrophages. A lower GLP-1 signaling pathway score derived from gene enrichment analysis was correlated with reduced immune cell infiltration, poorer patient survival rates, and lower responsiveness to immunotherapy across multiple cancer types (88). Clinical trials like SUSTAIN 6 and LEADER have evaluated the long-term cardiovascular and cancer-related effects of GLP-1 receptor agonists. The SUSTAIN 6 trial indicated a marginally elevated occurrence of LC associated with semaglutide in comparison to placebo (8/155 vs 6/139, HR 1.33, 95% CI: 0.46-3.82) (89), whereas the LEADER trial observed a reduction in LC events with liraglutide (28/470 vs 33/419; HR 0.85, 95% CI: 0.51-1.40) (90). The results indicate that the relationship between GLP-1 receptor agonists and LC risk is not yet fully understood, necessitating additional long-term research to draw conclusive insights.

### 5.3 Sodium-glucose cotransporter-2 inhibitors

**Figure 2:**
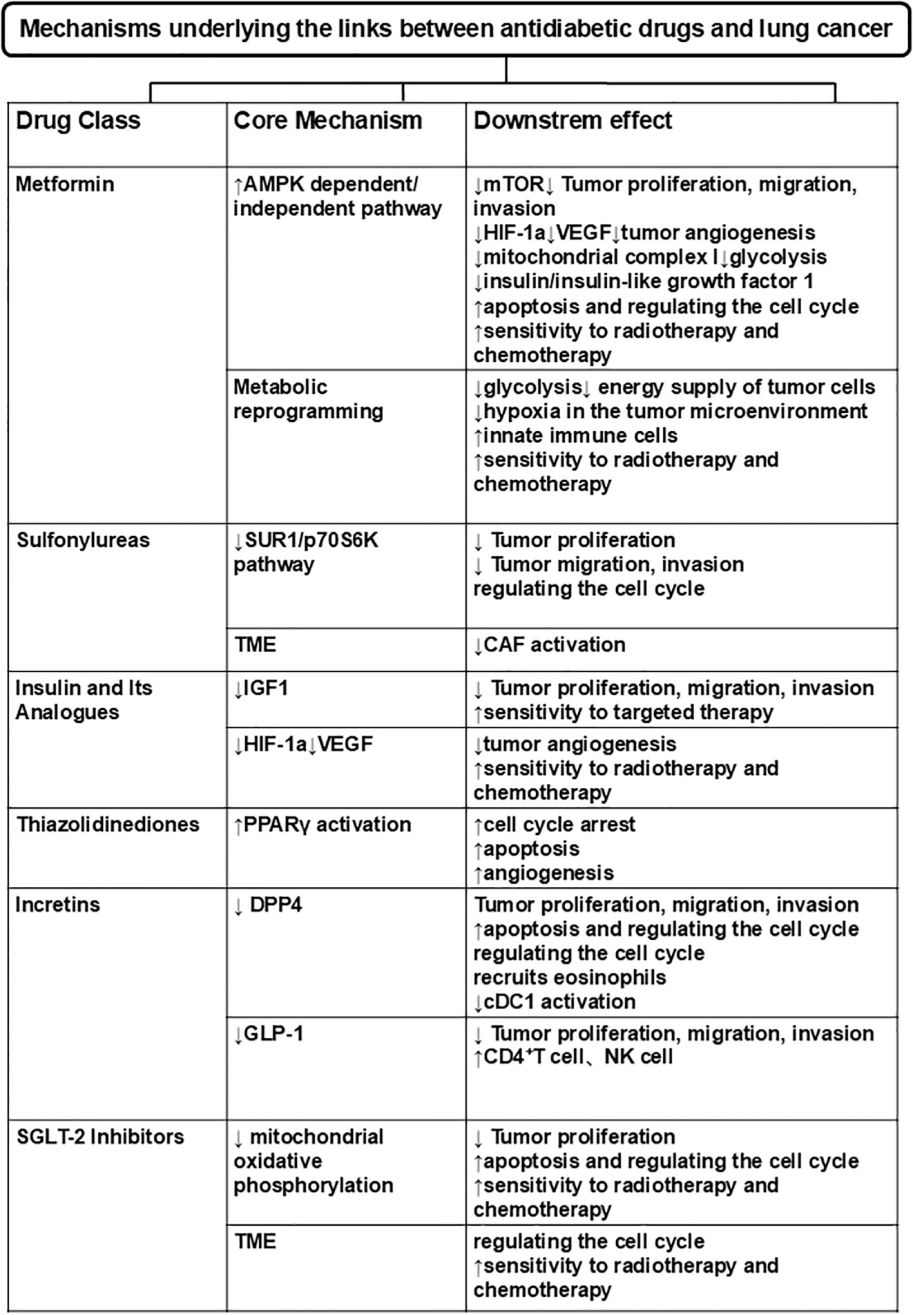
A summary diagram of mechanisms underlying the links between antidiabetic drugs and lung cancer.

Blood glucose levels are reduced by SGLT-2 inhibitors, a new class of anti-glycemic medications that reduce glucose reabsorption in the kidneys. SGLT-2 inhibitors are used to treat T2DM, either as monotherapy or in combination with other agents such as metformin, which has been approved for this indication (91). Recent research suggests that SGLT-2 inhibitors may possess anticancer (29) properties, as evidenced by findings such as diminished tumor growth and enhanced survival rates in early-stage LC mouse models (92), suppression of LC cell proliferation (29), and the promotion of apoptosis in drug-resistant NSCLC cell lines observed *in vitro* (93). Initial clinical findings indicate that SGLT-2 inhibitors could provide beneficial effects for individuals suffering from cancers affecting the gastrointestinal tract, lung, pancreas, prostate, and liver (94, 95). Linda et al. illustrated that canagliflozin impeded the proliferation of lung and prostate cancer cells mainly through the suppression of mitochondrial oxidative phosphorylation, consequently amplifying the effects of docetaxel and radiotherapy on tumor cell growth (29). Canagliflozin improves NSCLC response to chemotherapy through a core axis of mitochondrial inhibition-AMPK activation-mTOR-HIF-1α pathway blockade, combined with histone deacetylase 2 (HDAC2)-mediated epigenetic regulation and cell cycle modulation. It is a highly promising candidate drug for the combination therapy of NSCLC and further validates the repurposing value of SGLT2 inhibitors in the treatment of malignant tumors such as lung cancer (69). The reduction of glucose uptake by neoplastic cells constitutes a notable mechanism through which SGLT-2 inhibitors might demonstrate their anti-tumor properties (96). Observational studies indicate that the use of SGLT-2 inhibitors correlates with a reduced risk of LC and enhanced survival rates in patients with NSCLC (97). Summary of the mechanisms underlying the effects of different antidiabetic agents in lung cancer was shown in **Figure 2**.

## 6 Prospects and challenges

Given the chronic and progressive nature of T2DM, its management typically evolves through a staged approach, commencing with lifestyle modifications and advancing to various oral hypoglycemic agents before insulin therapy becomes necessary. Notably, these agents are unlikely to become first-line treatments for lung cancer, as current research primarily highlights potential beneficial mechanisms rather than definitive therapeutic effects.

Significant limitations in the design of existing clinical studies undermine the reliability of the evidence concerning the use of antidiabetic drugs in patients with coexisting diabetes and lung cancer. Most clinical studies are retrospective observational cohorts or meta−analyses prone to selection bias, immortal time bias, and confounding by glycemic control, comorbidities, and smoking status. Prospective trials are limited by small sample sizes, unstandardized dosing (e.g. metformin 500–2000 mg/day), undefined optimal time windows, and inconsistent endpoints, leading to high heterogeneity across studies. Preclinical research relies heavily on cell lines and xenografts that incompletely mimic the human tumor microenvironment, limiting translational value.

Confounding remains substantial: hyperglycemia, hyperinsulinemia, and chronic inflammation independently worsen lung cancer prognosis and may distort observed drug effects. Patient subgroups differ widely in histology, driver mutations, disease stage, and baseline metabolism, contributing to contradictory outcomes, especially between EGFR−mutant and wild−type NSCLC.

Critical evidence gaps persist: no validated predictive biomarkers identify patients likely to benefit; optimal dosing, timing, and duration with chemotherapy, targeted therapy, or immunotherapy remain undefined; long−safety and drug–drug interaction data are scarce; and head−to−head comparisons of antidiabetic drug classes are lacking. Mechanistic crosstalk between metabolic pathways and oncogenic signaling requires further clarification.

Nevertheless, antidiabetic agents hold considerable promise. Metformin, SGLT−2 inhibitors, and TZDs exert consistent anti−tumor effects via AMPK/mTOR modulation, metabolic reprogramming, immune activation, and angiogenesis inhibition. These drugs are affordable, widely accessible, and have well−established safety profiles supporting their potential as complementary strategies rather than first-line treatments for lung cancer.

Future research demands large, multicenter, randomized controlled trials with standardized protocols, biomarker stratification, and long−term follow−up. Mechanistic studies using patient−derived models and integrated multi−omics will refine combination strategies. With rigorous validation, metabolic targeting could become a practical, cost−effective adjuvant approach to improve lung cancer outcomes.
